# Controlling the Size of Hydrotalcite Particles and Its Impact on the Thermal Insulation Capabilities of Coatings

**DOI:** 10.3390/ma17092046

**Published:** 2024-04-26

**Authors:** Yanhua Zhao, Guanhua Shen, Yongli Wang, Xiangying Hao, Huining Li

**Affiliations:** 1 Guangdong Provincial Key Laboratory of Environmental Health and Land Resource, College of Environmental and Chemical Engineering, Zhaoqing University, Zhaoqing 526061, China; 2017010008@zqu.edu.cn (Y.Z.); 2021010065@zqu.edu.cn (Y.W.); 2 Zhaoqing Environmental Functional Materials Engineering Technology Center, College of Environmental and Chemical Engineering, Zhaoqing University, Zhaoqing 526061, China; 3 Zhaoqing Rivers High-Tech Materials Co., Ltd., Zhaoqing 526061, China; 13902398773@163.com

**Keywords:** Mg-Al layered double hydroxides, particle size regulation, thermal insulation, thermal conductivity

## Abstract

This study focuses on the development of high-performance insulation materials to address the critical issue of reducing building energy consumption. Magnesium–aluminum layered double hydroxides (LDHs), known for their distinctive layered structure featuring positively charged brucite-like layers and an interlayer space, have been identified as promising candidates for insulation applications. Building upon previous research, which demonstrated the enhanced thermal insulation properties of methyl trimethoxysilane (MTS) functionalized LDHs synthesized through a one-step in situ hydrothermal method, this work delves into the systematic exploration of particle size regulation and its consequential effects on the thermal insulation performance of coatings. Our findings indicate a direct correlation between the dosage of MTS and the particle size of LDHs, with an optimal dosage of 4 wt% MTS yielding LDHs that exhibit a tightly interconnected hydrotalcite lamellar structure. This specific modification resulted in the most significant improvement in thermal insulation, achieving a temperature difference of approximately 25.5 °C. Furthermore, to gain a deeper understanding of the thermal insulation mechanism of MTS-modified LDHs, we conducted a thorough characterization of their UV-visible diffuse reflectance and thermal conductivity. This research contributes to the advancement of LDH-based materials for use in thermal insulation applications, offering a sustainable solution to energy conservation in the built environment.

## 1. Introduction

With increasing energy scarcity nationwide, the collective focus on energy conservation and reducing consumption has become a societal priority. In construction, using insulating materials to minimize energy loss in buildings is crucial [1,2,3,4,5]. Retrofitting buildings to optimize energy usage is essential for sustainable development. While adding thermal insulating materials is a common approach, current energy-saving retrofitting methods often overlook the environmental impact. Traditional inorganic insulation materials like asbestos, mineral wool, and expanded perlite offer good thermal properties but harm the environment [6]. Organic alternatives like polymer foam and foam asbestos insulation materials have drawbacks such as high volume and associated costs. Consequently, innovative insulation materials have been developed to improve energy conservation in both existing and new constructions [7,8,9]. These materials offer enhanced thermal insulation properties, possess economic viability, and support carbon neutrality. Developing high-performance insulation materials to reduce building energy consumption is crucial [10].

The widespread use of heating and cooling systems in high-energy-consuming global constructions leads to increased energy wastage. However, applying water-based thermal insulation coatings on surfaces can effectively provide cooling and heat-insulating effects [11]. This type of coating uses water as a dispersion medium, making it eco-friendly and non-toxic, which helps achieve energy conservation and emission reduction goals. Thermal insulation coatings are typically categorized into barrier, reflective, radiation, and composite types based on their heat insulation mechanisms. Reflective coatings in particular can impede heat conduction by relying on the surface reflectivity of the coating, which are fabricated using a variety of materials and are applied to solar cells, modules, and other contemporary applications [12,13,14,15,16,17]. Various factors influence the reflectivity of the coating, such as the ratio between the filler refractive coefficient and the coating resin matrix, coating film thickness, particle size, and distribution of the filler [18,19,20].

Aluminum layered hydroxides, also known as layered double hydroxides (LDHs) or hydrotalcite-like compounds, have found applications in insulation materials due to their unique properties, e.g., a layered structure with positively charged brucite-like layers and interlayer anions, providing them with excellent thermal and flame-retardant properties [21,22,23,24,25,26,27]. The low specific gravity and thermal conductivity of LDHs make them suitable for insulating barrier coatings. LDHs exhibit outstanding thermal insulation, UV reflection, infrared absorption, and scattering capabilities. Post-calcination at a specific temperature can enhance LDHs’ insulation and render them ideal as reflective materials, which underscores LDHs’ potential for broad applications in water-based insulation materials [23]. As a cost-effective mineral, hydrotalcites offer diverse manufacturing options, and abundant natural deposits also position hydrotalcites favorably for construction applications, either as a building material or a coating filler [28,29,30]. Sun et al. demonstrated hydrotalcites’ ability to impact interior wall coating thermal performance by 5 °C through infrared blockage. However, limited data exist on hydrotalcites’ use in insulation coating formulations [12].

In our previous study, we found that using a one-step in situ hydrothermal method to modify LDHs with methyl trimethoxy siloxane (MTS) resulted in the largest particle size and distribution, leading to the most effective thermal insulation [31]. The research into controlling the size of LDH particles could significantly impact this field by optimizing the thermal insulation capabilities of these coatings, which is critical because the thermal behavior and properties of hydrotalcites, including their thermal stability and fireproofing performance, are influenced by their particle size [32,33,34]. Furthermore, we incorporated the MTS-modified LDHs as fillers in waterborne coatings to assess and compare their reflectivity, thermal insulation, and heat retention capabilities. Through comprehensive characterizations of both LDHs and coatings, we delved into the mechanisms behind thermal insulation and heat preservation.

## 2. Experimental

### 2.1. Materials

Raw materials such as sodium carbonate, sodium hydroxide, magnesium nitrate hexahydrate, MTS, film-forming additive, and aluminum nitrate nonahydrate were sourced from Guangzhou Chemical Reagent Factory Ltd. (Guangzhou, China), while the acrylic resin was obtained from Zhao Qing Rivers High-Tech Materials Co., Ltd. (Zhaoqing, China).

### 2.2. Preparation of MTS-Modified LDHs via One-Step In Situ Hydrothermal Method

Utilizing a one-step in situ hydrothermal synthesis approach as per our previous research, [27] the MTS-modified LDHs were produced. A standard protocol involved the dissolution of 19.2 g of Magnesium Nitrate Hexahydrate Mg(NO_3_)_2_·6H_2_O and 10.6 g of Al(NO_3_)_3_·9H_2_O in 160 mL of distilled water to create Solution C. Concurrently, Solution D was formulated by mixing approximately 8 g of NaOH and 8.6 g of Na_2_CO_3_ in 60 mL of distilled water. In addition, an aqueous solution of MTS with a pH level of 4–5, known as Solution E, was prepared by dissolving a specific quantity of MTS (1.0 g, equivalent to 1 weight percent relative to the LDH) in 10 mL of distilled water.

Subsequently, Solution D was incrementally introduced into a mixture of Solutions C and E with robust agitation maintained at an ambient temperature. The ensuing blend was poured into a hydrothermal autoclave for processing, employing the same stirring technique as was used for the control group. The precipitate that formed was then isolated via filtration, thoroughly rinsed with distilled water, and subsequently subjected to drying at a temperature of 90 °C for a duration of 12 h. The outcome was a white solid product which was designated as LDHn (where n ranges from 0 to 5), with “n” denoting the weight percentage of MTS incorporated into the LDH.

### 2.3. Preparation of Thermal Insulating Coating Samples

The thermal insulating coating was prepared following the recipe outlined in Table 1. In the coating sample preparation, pre-measured acrylic resin and a film-forming additive were combined per the formulation provided in Table 1. This blend was stirred gently for approximately 6 min at a low speed to ensure uniform distribution. Next, the filler and water were slowly added to the mixture, followed by vigorous stirring for an additional 20–35 min at 1500 r/min to achieve homogeneous dispersion. Finally, the coating samples were completed by adding a specified quantity of curing agent.

### 2.4. Preparation of Thermal Insulating Coating Films

The coating film was crafted in accordance with the guidelines specified in the GB/T1765-1979 standard [35]. To fabricate thermal insulating films, the pre-prepared coating samples were evenly applied onto an asbestos substrate. This application process was facilitated by using a 150 µm wire rod to distribute the coating uniformly across the surface of the asbestos sheet. Afterward, the coated samples underwent a drying phase, following which their thermal insulation performance was evaluated.

### 2.5. Characterizations

The Powder XRD patterns of the LDHs were obtained using a Bruker D8 Advance diffractometer with Cu Kα radiation (k = 1.5406°) at 40 kV and 40 mA, scanning in the 2θ range of 5–70° at a rate of 3°/min. Fourier Transform Infrared (FT-IR) spectra were captured with an infrared spectrometer (IRTracer-100, Shimazu, Tokyo, Japan) in the 400–4000 cm^−1^ spectral range, employing the KBr pellet technique at a resolution of 4 cm^−1^. Morphological observations were performed with a scanning electron microscope (SUPRA55, Zeiss, Jena, Germany) at 30 kV and 10.0 μA. UV-Vis diffuse reflectance spectra were recorded using a spectrophotometer with an integrating sphere (UV-2600, Shimadzu, Japan), with BaSO_4_ as the reference standard. Particle size distributions were analyzed using a laser granulometer (LA-960S2, HORIBA Ltd., Osaka, Japan), and thermal conductivities were determined via the transient plane source method using Hot Disk TPS 2500S (Hot Disk AB, Göteborg, Sweden) thermal conductivity measurement equipment.

### 2.6. Thermal Insulation Performance Evaluation

**Figure 1:**
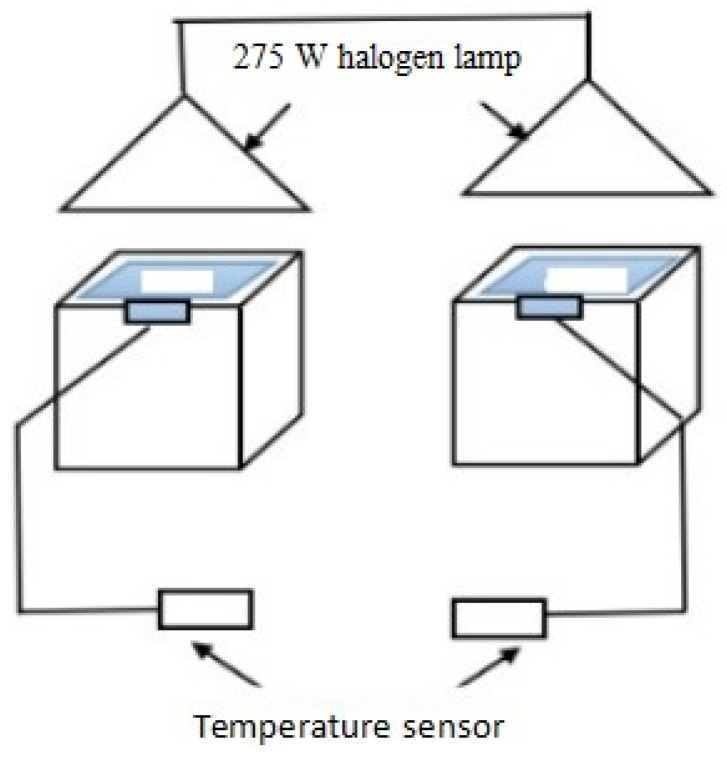
Schematic illustration of the custom-built thermal insulation testing device.

The film samples’ thermal insulation performance was assessed using a customized thermal insulation testing apparatus, illustrated in Figure 1 [31]. The evaluation involved exposing the coating samples to a 275 W halogen lamp as the light source on a flat asbestos fiber cement panel. Temperature sensors recorded the backside temperature of each asbestos slab every 5 min during a 45 min test period. The thermal insulation effectiveness of the coating samples was determined by comparing the temperature of an uncoated asbestos sheet (T_blank_) to that of an asbestos sheet coated with the film sample (T_film_). The thermal insulation performance was quantified by calculating the thermal insulation temperature difference, ΔT = T_blank_ − T_film_.

## 3. Results and Discussion

### 3.1. Structure Characterizations

**Figure 2:**
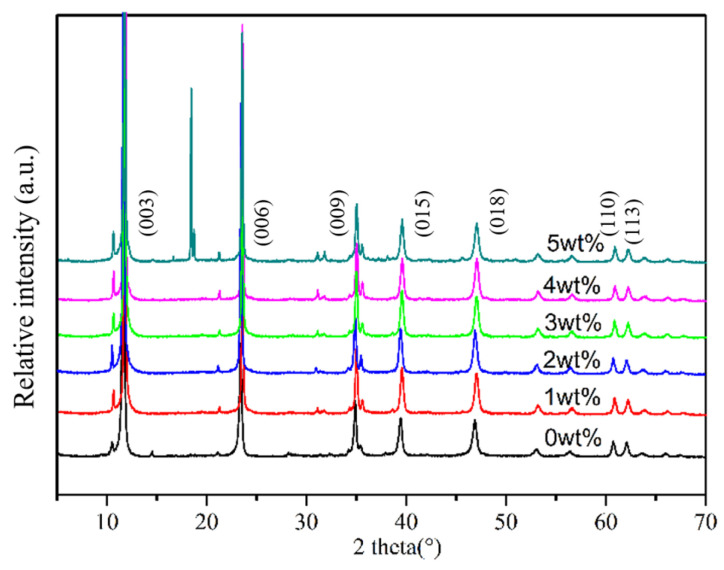
XRD patterns of LDHs prepared with different amounts of MTS (from up to down: 5, 4, 3, 2, 1, 0 wt%).

Figure 2 illustrates XRD patterns of the obtained LDHs with varying amounts of MTS (from up to down: 5, 4, 3, 2, 1, 0 wt%). These patterns exhibit a well-ordered layer structure with a basal spacing (d_003_) of 7.9 Å, corresponding to CO_3_^2−^ (Mg-Al-CO_3_^2−^-LDH), in alignment with standard ICDD reference pattern 00-022-0700 (hydrotalcite, syn-Mg_6_A_l2_(OH)_16_CO_3_·3H_2_O) [36,37]. The LDHs show narrow, sharp patterns with high diffraction intensity within the 2θ range of 7–37° (d_003_, d_006_, and d_009_), as well as similar d_00l_ values to the original LDH. Additionally, symmetrical lower reflections within the 2θ range of 60 to 63° (d_110_ and d_113_) indicate high crystallinity of the resulting LDHs. The consistent d-spacings suggest minimal impact on the LDH chemical structures from both modification methods. The peaks occur at d_003_, d_006_, and d_009_ of the modified LDH shift compared to the pristine LDH due to MTS insertion into LDH basal spacing. The one-step in situ method does not significantly alter the LDH structure, as surface reactions between MTS and LDH occur without MTS insertion into the interlayer of LDH. External MTS on LDH decreases viscosity, enhancing LDH crystallinity (higher than original LDH), which is valuable for modified LDH practical applications in nanocomposite materials. However, with the addition of 5 wt% MTS during preparation, two distinct peaks emerged in the 2θ range from 18.4 to 18.6°, demonstrating a noticeable deviation from other samples and suggesting a structural alteration in the LDH. Consequently, the LDH prepared with 5 wt% of MTS was excluded from subsequent studies.

**Figure 3:**
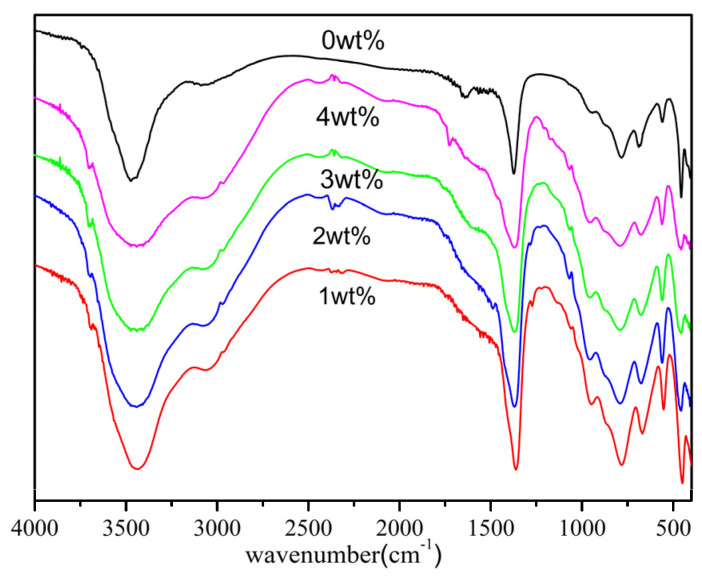
FT-IR spectra of LDHs prepared with different amounts of MTS (from up to down: 0, 4, 3, 2, 1 wt%).

Figure 3 presents the FT-IR spectroscopic data for LDHs synthesized with different concentrations of MTS, arranged from highest to lowest (0, 4, 3, 2, 1 wt%). In the spectrum of the unmodified LDH, bands associated with hydroxyl groups (-OH) are observed at 3466 cm^−1^, corresponding to water molecules, and at 3073 cm^−1^, which is indicative of a bridge bond between water and anions. Additionally, a bending vibration mode related to water is detected at around 1636 cm^−1^. A distinct peak at approximately 1350 cm^−1^ is attributed to the asymmetric stretching vibration of the carbonate ion (CO_3_^2−^), whereas the band at 827 cm^−1^ is associated with the v_2_ vibrational mode of CO_3_^2−^ ions located within the intercalated spaces of the LDH layers [38]. The presence of bands at 576 cm^−1^ is indicative of the metal–oxygen (M–O) stretching vibration, where M represents magnesium (Mg^2+^) or aluminum (Al^3+^) ions. The FT-IR spectra of the MTS-modified LDHs closely resemble those of the original LDH, indicating that the structural integrity of the LDH framework was preserved despite the modification process, a finding that is consistent with XRD results. Furthermore, an additional albeit weak band appears at about 1060 cm^−1^ in the spectra of LDHs treated with MTS, which is characteristic of the Si-O-Si vibrational mode unique to MTS. The FT-IR spectra provide clear evidence of the successful functionalization of LDHs with MTS.

**Figure 4:**
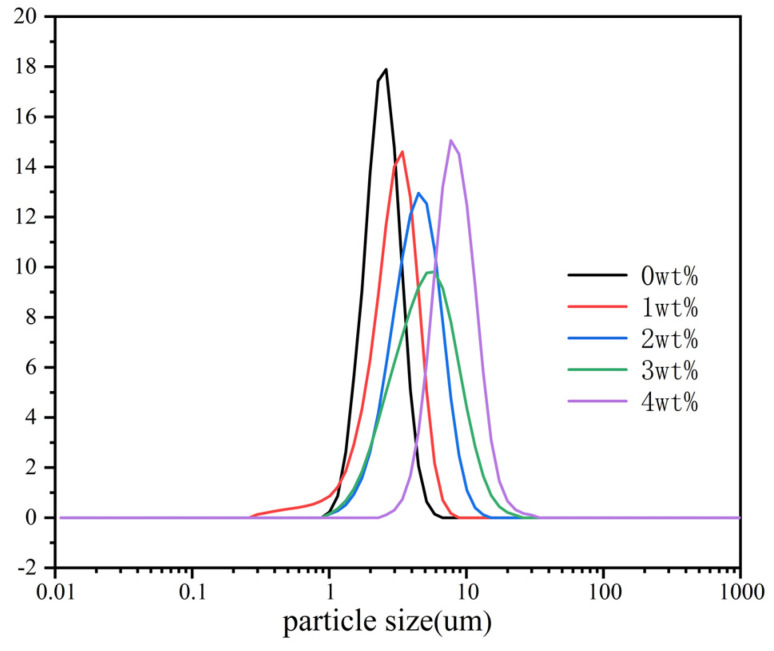
Particle size distribution curves of LDHs prepared with different amounts of MTS (from left to right: 0, 1, 2, 3, 4 wt%).

**Figure 5:**
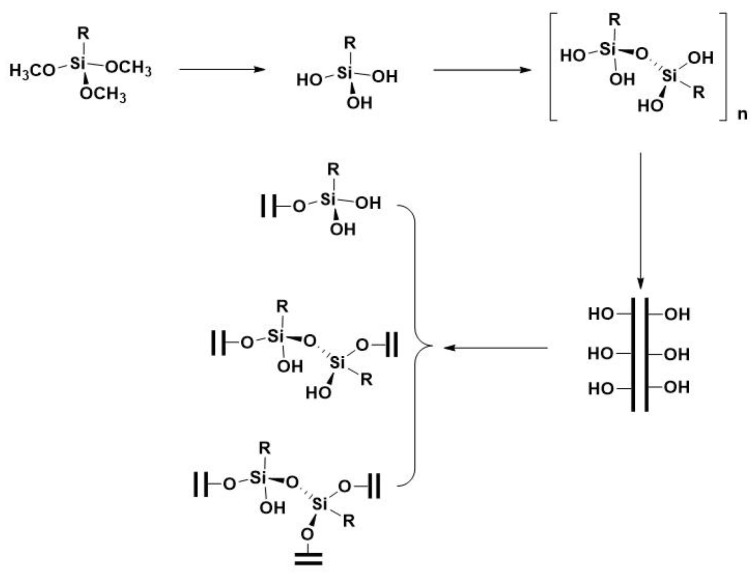
Illustration of the mechanism of LDHs formation.

Acknowledging the pivotal role that particle size and distribution play in determining the thermal insulation characteristics of the end product, meticulous adjustments were made to the quantities of MTS to achieve precise control over the particle size and distribution of the MTS-modified LDHs. Figure 4 illustrates the particle size distribution curves of LDHs prepared with varying MTS quantities (from left to right: 0, 1, 2, 3, 4 wt%). The native LDH exhibits a rather narrow particle size distribution, with particles averaging around 2.4 μm in diameter. Notably, as the concentration of MTS increases, there is a marked escalation in both the particle size and distribution of the MTS-modified LDHs. This enlargement is believed to result from the interaction between MTS and the hydroxyl groups present on the surface of the LDH layers. The MTS molecules bind to the LDH crystals, facilitating the formation of a polysiloxane network that establishes an intermolecular Si-O-Si bond matrix linking adjacent silylated LDH particles (as depicted in Figure 5, where R denotes a methyl group) [18,39]. The LDH sample synthesized with 4 wt% MTS exhibited the largest particle size, showcasing a broad range of sizes with an average of 9.8 μm. Thus, in subsequent thermal insulation performance evaluations, we could analyze how particle size and distribution affect thermal insulation effectiveness.

**Figure 6:**
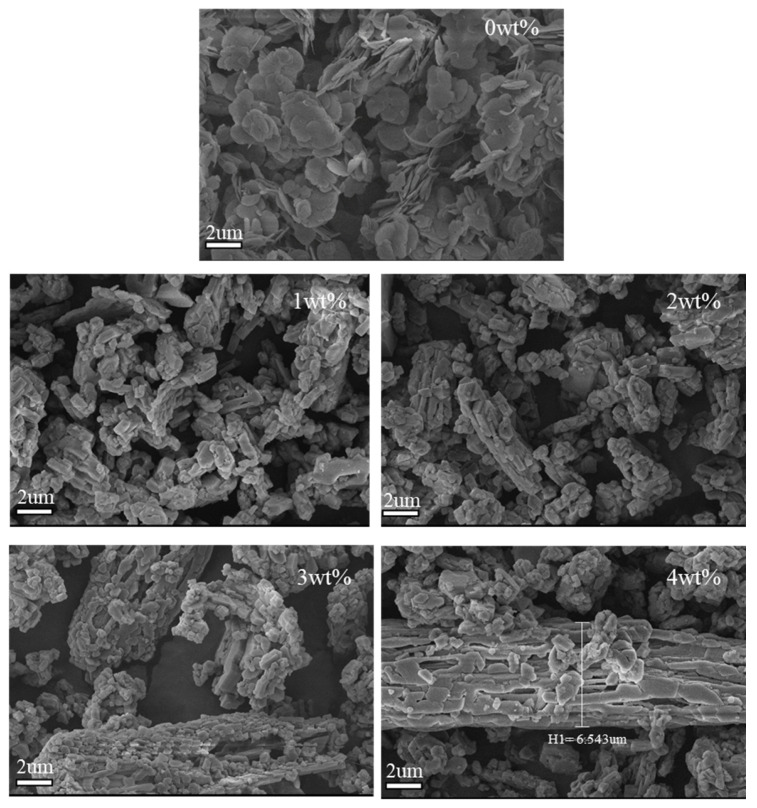
SEM images of LDHs prepared with different amounts of MTS (0, 1, 2, 3, 4 wt%).

Figure 6 shows SEM images of LDHs prepared with different amounts of MTS (0, 1, 2, 3, 4 wt%). In Figure 5, the LDH sample containing 0 wt% MTS forms particles with a hexagonal lamellar structure, featuring rounded corners. These particles measure approximately 2 μm, aligning with the particle size measurement depicted in Figure 4. LDHs prepared with MTS modification exhibit a unique strip-shaped structure that tightly aggregates to form a rod-like polymer. Increasing the MTS amount leads to thicker rod-shaped LDH layers due to cross-linking reactions within MTS, its hydrolysis products, and hydroxyl groups on the LDH layers. This process connects the LDH sheet layers more tightly. The enhanced cross-linking with higher MTS levels results in larger LDH particle sizes and tighter LDH sheet layers that strengthen the barrier against UV-visible and near-infrared light, enhancing film reflectivity and insulation effectiveness. These findings align with the particle size and distribution tests, showing that varying MTS amounts influence LDH morphology and particle size.

### 3.2. Thermal Insulation Properties and UV-NIR Reflectance

#### 3.2.1. Thermal Insulation Properties

**Figure 7:**
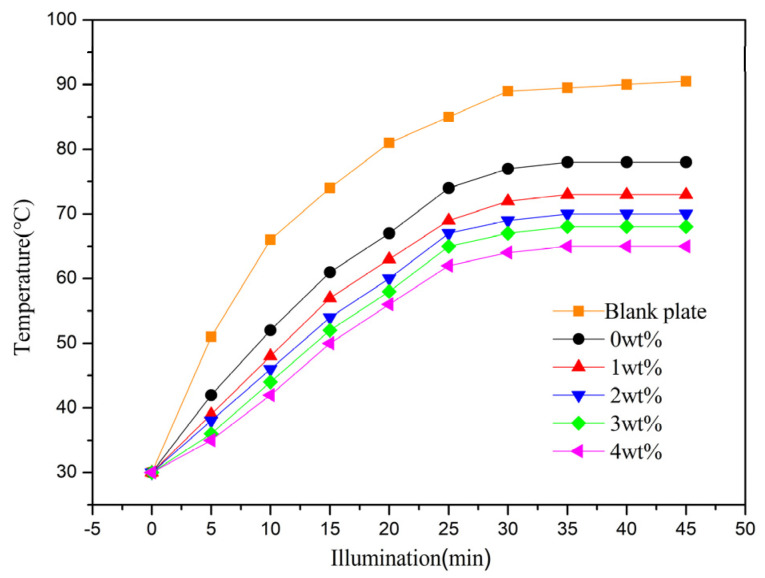
The T_eq_ variations of the plates with coatings containing LDHs prepared with different amounts of MTS (from up to down: blank plate, 0, 1, 2, 3, 4 wt%) are plotted as a function of time.

The thermal insulation properties of LDH-coated films were assessed using a custom-built testing device, illustrated in Figure 7. The tests began at room temperature and continued until equilibrium temperatures (T_eq,_ defined as minimal temperature fluctuations (<1 °C) over a 5 min period) were achieved. All tests were conducted under consistent lighting conditions, starting from an initial temperature of 30 °C [14]. Temperature fluctuations were monitored at the rear surface of cement reference panels coated with thermal insulation films, with an LDH-free coating serving as the control. Without thermal insulation coatings, the blank panel temperature swiftly rose under iodine–tungsten light, reaching a T_eq_ value of 89.4 °C in about 30 min. Conversely, panels coated with LDH-based thermal insulation films displayed reduced back surface temperatures, indicating superior thermal insulation. Notably, T_eq_ values decreased with increasing MTS content. Among the LDH-coated films, the 4 wt% MTS-modified LDH coating exhibited the best thermal insulation performance, with a T_eq_ value of approximately 61 °C and a slower temperature increase rate. These findings underscore the efficacy of MTS-modified LDH fillers for thermal insulation applications.

A significant observation from the thermal insulation tests is the substantial influence that larger particle sizes and broader distributions have on the effectiveness of thermal insulation in coatings that incorporate LDHs [40]. The thermal conductivity of a composite material is predominantly determined by the conductivities of its two main components, the continuous and the dispersed phases. In this context, we introduced a modified LDH as the dispersed phase, which was then combined with a cured polyacrylic resin acting as the continuous phase. The fillers with smaller particle sizes are expected to establish a more interconnected network within the material, leading to a notable reduction in thermal conductivity and enhanced insulation characteristics. This observation underscores the critical role of precise filler particle size control in crafting high-performance thermal insulation coatings. Table 2 shows the heat-insulation performance of the coatings with different LDH fillers. The thermal insulation temperature difference for hydrotalcite synthesized without MTS addition is 12.5 °C. As the MTS amount increases, the thermal insulation effect of the LDH coating film improves progressively. For instance, LDH synthesized with 4 wt% MTS demonstrates a thermal insulation temperature difference of 25.5 °C, highlighting the significant impact of MTS content on enhancing LDH’s thermal insulation properties.

#### 3.2.2. UV-NIR Reflectance

**Figure 8:**
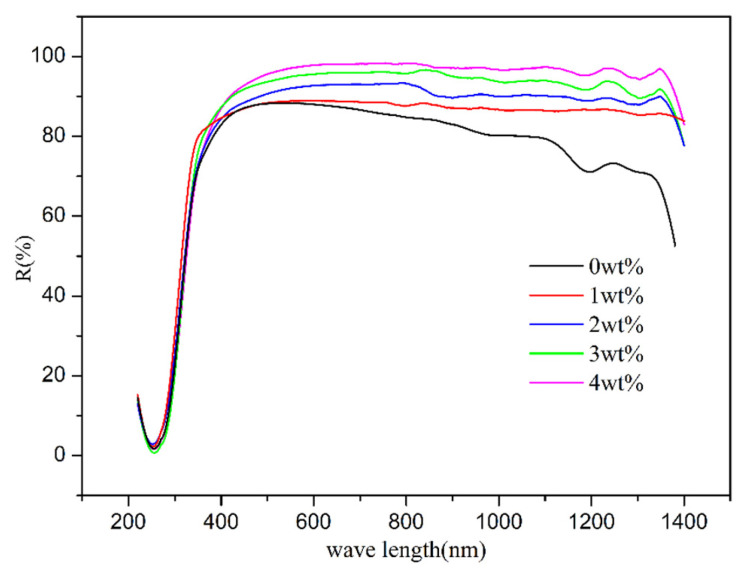
UV-NIR spectral reflections of the coatings containing different LDHs prepared with different amounts of MTS (from up to down: 0, 1, 2, 3, 4 wt%) as a function of wavelength.

To explore the thermal insulation mechanism, UV-NIR diffuse reflection analysis was performed on coating films containing various LDH fillers. Figure 8 shows the UV-NIR spectral reflections of the coatings containing different LDHs prepared with different amounts of MTS (up to down: 0, 1, 2, 3, 4 wt%) as a function of wavelength. The UV-NIR spectral reflections of the coatings containing MTS-modified LDHs are noticeably higher compared to those of the unmodified sample. Furthermore, the UV-NIR spectral reflections of the coatings with MTS-modified LDHs rise as the MTS content increases. This can be linked to larger particle sizes and distributions, as well as the formation of densely packed layers, as observed in the particle size and SEM analyses. Consequently, incorporating LDH into the coating system enhances the reflectivity of the coated plate. The particle size of LDH is deemed crucial in determining the efficiency of UV-NIR light reflection. The heightened UV-NIR reflectivity of the coating is likely due to an enlargement in the particle size of the incorporated LDH sample. Fortunately, the particle size and its distributions can be effectively controlled by adjusting the MTS content in the one-step in situ LDH preparation process.

**Figure 9:**
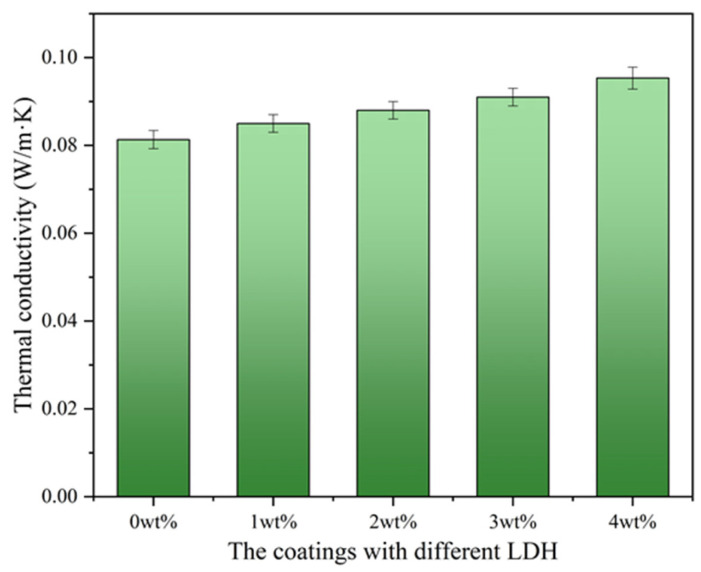
Thermal conductivity of the coatings containing different LDHs prepared with different amounts of MTS (from left to right: 0, 1, 2, 3, 4 wt%).

Thermal conductivity is a critical parameter when evaluating the thermal insulation properties of a material. It measures the ability of a material to conduct heat, indicating how effectively it can resist the transfer of thermal energy. Materials with low thermal conductivity are excellent insulators, as they impede the flow of heat, helping to maintain consistent temperatures and reduce energy consumption [41,42]. Against this background, the thermal conductivity of the coatings containing different LDHs prepared with different amounts of MTS (from left to right: 0, 1, 2, 3, 4 wt%) was also investigated here (Figure 9). The thermal conductivity of the coatings rises as the MTS content increases in the LDH preparation process. This trend is likely attributed to the cross-linking and stacking between LDH flake layers, along with an increase in particle size, which diminishes porosity between fillers and boosts filler density, subsequently elevating thermal conductivity [42,43]. Nonetheless, the variation in the thermal conductivity of hydrotalcite with diverse particle sizes is minimal, possibly because the layer spacing of hydrotalcite remains largely unchanged pre- and post-modification. Consequently, based on the thermal insulation effect, it can be deduced that thermal conductivity is not the primary factor influencing thermal insulation, and the low thermal conductivity of hydrotalcite makes it a promising filler for thermal insulation materials.

## 4. Conclusions

Concisely, the dimensions and distribution of LDHs can be readily manipulated by varying the MTS concentration in a single-step, in situ hydrothermal synthesis process, leading to a proportional growth in particle size as the MTS content increases. The effectiveness of the synthesis and modification processes was confirmed through FT-IR analysis, and XRD studies indicate that the crystalline integrity of the LDHs is preserved when the MTS concentration does not exceed 5 wt%. SEM examination confirms the particle size measurements and observes that LDHs tend to form clumps, yet they maintain a relatively uniform and orderly stratified structure, creating “rod-like” formations. When LDHs are employed as additives in the formulation of thermal insulation coatings, they impart different levels of thermal insulation efficacy. Notably, the coating that incorporates LDH modified with 4 wt% MTS achieves the highest thermal insulation performance, with a surface temperature (T_eq_) of 61 °C, which is a significant reduction of 28.4 °C compared to the untreated control surface. Additionally, the UV-NIR spectral reflectance of coatings with MTS-modified LDH is superior to those with non-modified LDH. The thermal conductivity of coatings containing MTS-modified LDH is also found to be lower than those with unmodified LDH, and this advantage increases with higher MTS concentrations. This study underscores the potential of LDH materials in advancing thermal insulation technology.

## Figures and Tables

**Table 1:** Formulation of the thermal insulating coatings.

Ingredients	wt (%)
Acrylic resin	32
Film-forming additive	7
Fillers	45
Water	12
Curing agent	3

**Table 2:** Heat-insulation performance of the coatings with different LDH fillers.

MTS (wt%)	0	1	2	3	4
Heat-insulation temperature Difference ΔT (°C)	12.5	0	23	24	25.55

## Data Availability

Data are contained within the article.
